# Sussex County Hospital

**Published:** 1923-04

**Authors:** 


### SUSSEX COUNTY HOSPITAL.

### REPORT OF THE INQUIRY COMMITTEE.

FROM the Report of the Committee of Inquiry which was appointed last October "to consider and advise as to obtaining fresh sources of income and increased economy of administration," we take the following passages. The Committee of the Sussex County Hospital at Brighton, of which the Rev. E. D. L. Harvey, O.B.E., was chairman, included Dame Alice Godman, D.B.E., and the Hon. Sir Arthur Stanley, G.B.E. Mr. J. Harold Penfold was the honorary secretary. The Committee's first step was to obtain a full investigation of the administrative and financial position of the Hospital from Sir Napier Burnett, to whom they are most grateful for his exhaustive and valuable report. The Committee's recommendations were unanimous. In a covering letter, Mr. Harvey writes: "While possibly some exception may be taken to the figures in connection with the cost per bed, the Committee feel that this does not in any way affect their main conclusions. . . . The Committee feel that the board failed to adapt their methods of administration to the changed conditions brought about by the war, and that the only hope of securing more economical administration is to secure more efficient control of expenditure by the Superintendent, he being in fact the Chief Executive Officer with complete control over the expenditure of every department."

### Expenditure.

The Committee express their regret that the Board of Management, on finding that their expenditure in 1921 greatly exceeded that of 1920, in spite of the fact that there had been a considerable fall in nearly all prices, and that it more and more largely exceeded their income, did not themselves call in an expert, but simply appealed to the public for more funds and finally closed over 100 beds :—

From his examination of the accounts of the County Hospital, Sir Napier Burnett reports that under two heads of expenditure alone there is a very large excess over the average of comparable hospitals. Under the head of Domestic the expenditure of the County exceeds the group average by 32 per cent., or £72 14s. per bed, as against an average of £62 13s. per bed, for the two years 1920 and 1921 taken together. Under the head of Surgery and Dispensary the County expenditure is 38 per cent. above the group average, or £62 12s. per bed, as against £49 14s. per bed for the same period. Under these two heads alone we find an expenditure of £22 19s. per bed above the group average of comparable hospitals, and in the opinion of the Committee this great difference is, to a large extent, due to lack of proper supervision.

### Surgery and Dispensary.

The Committee are of opinion that the food is on the whole good, but a large percentage of patients are on special diet, which adds to the cost. With regard to the surgery and dispensary, which shows the largest excess of expenditure over the average, there is apparently, in the dispensary at least, great lack of control :—

"It appears that the pharmacist buys when and where he thinks best, and although requisitions are put before the Drug Committee, and passed by them, more often than not this is only a confirmation of what the pharmacist has already done. More striking still is the apparent lack of control and record of the issue of drugs, chemicals, disinfectants, &c."

### Few Workmen's Subscriptions.

According to some details given in his report by Sir Napier Burnett as to the income of a certain group of hospitals, it appears "that the Sussex Hospital depends for nearly 40 per cent. of its income, as against an average of 16 per cent. in the other hospitals of the group, on what are called donations—the term including, besides gifts usually so called, the proceeds of bazaars, special appeals and other non-recurring contributions" :—

"It will be admitted that this cannot be considered a healthy feature. It is no doubt true 'that the figure in which Brighton stands out above its fellow hospitals, namely, donations, reflects considerable credit upon the energy of its appeal department,' but these constant special appeals involve an enormous tax on the energy of the collectors, undoubtedly weary the charitable public, and seriously affect similar institutions who are driven to exploit the same field. We realise that it is only by these means that the hospital has been enabled to continue its work to the extent that it has, but the Board of Management must be desirous of seeing the bulk of its income derived from dependable instead of casual resources. The directions in which it is clearly driven to look are subscriptions and patients' and workmen's contributions. The former in the Royal Sussex County Hospital form but 8 per cent. of the total, as against an average of 14 per cent. for the Group. No doubt the attempt to obtain permanent subscriptions from the county and towns will be far from easy."

### How to Raise Money.

The Committee suggest that such a campaign should be undertaken in combination with other similar institutions. Sir Napier Burnett devotes considerable space to the consideration of various means for obtaining more contributions from workmen :—

"In Sussex there is a scheme in existence — which is known as the Sussex Provident Scheme — and though it is criticised on various grounds by some of the hospital witnesses, it would obviously be unwise, if not impracticable now, to start a competing scheme. It provides for an insurance by groups of workmen by subscriptions of 3d. per week, which entitles to hospital benefits for each subscribing member and his family from any affiliated hospital. All, or nearly all, the Brighton hospitals are affiliated, and it is understood that the Committee of the scheme pay the whole cost of each bed occupied by their members, and that any surplus is available for division at the discretion of the Committee."

Finally, the Committee "commend their report to the serious consideration of the Board of Management, assuring them that it has been drawn up with great care after full investigation and with an earnest desire that it may lead to a system of administration of the County Hospital which will revive the confidence of the subscribers and general public."

### An Improved Situation.

Notwithstanding these difficulties the finance of the Hospital has improved. The debt of £12,000 at the beginning of 1922 has been converted, says the annual report, into a credit balance of £5,000. The Voluntary Hospitals Commission has promised a grant of £3,950 on condition that 50 beds be reopened by March 31, and the remainder not later than September.

